# Computational analysis of visible frequency plasmonic properties of graphene on wide band gap heterostructures

**DOI:** 10.1038/s41598-026-40039-y

**Published:** 2026-02-15

**Authors:** Muhammad Qamar, Ghulam Abbas, Meiyong Liao, Satoshi Koizumi, Takatoshi Yamada, Bohuslav Rezek

**Affiliations:** 1 Faculty of Electrical Engineering, Czech Technical University in Prague, Technická 2, Prague, 16627 Czechia; 2 National Institute for Materials Science (NIMS), 1-1 Namiki, Tsukuba, 305-0044 Japan; 3 National Institute of Advanced Industrial Science and Technology (AIST), Central 5, 1-1-1 Higashi, Tsukuba, 305-8565 Japan

**Keywords:** Graphene, Boron nitride, Silicon, Diamond, Plasmonic properties, Electric field simulations, Materials science, Nanoscience and technology, Optics and photonics, Physics

## Abstract

Control over plasmonic properties and local electric field enhancement has become an essential aspect of many modern technologies. Here we investigate these phenomena in graphene / hexagonal boron nitride (G/h-BN) heterostructures positioned on silicon (Si) and silicon dioxide (SiO_2_) substrates. Using finite element method for physics-based simulations of radio-frequency (RF) fields in optical range, we analyze electric field at the edges, on the flakes, and in the surrounding regions of the G/h-BN heterostructures. The results demonstrate that the electric field distribution around and within the heterostructure is strongly dependent on the thickness of graphene and h-BN flakes. The highest electric field amplification and focusing occurs at the G/h-BN edge for h-BN thicknesses between 80 and 100 nm on the Si substrate. In contrast, the SiO_2_ substrate substantially reduces overall field intensity in the G/h-BN heterostructures in comparison to the Si and reference structure without h-BN. These findings provide a consistent theoretical explanation for previously reported experimental Raman spectroscopy data on G/h-BN heterostructures and corroborate the model of localized charge carrier accumulation at the nanoscale G/h-BN edges on Si substrates. Furthermore, the study provides predictions for optimal excitation frequencies and for tailoring graphene plasmonic features in visible spectral range with the use of diamond and other CMOS compatible materials.

**Supplementary Information:**

The online version contains supplementary material available at 10.1038/s41598-026-40039-y.

## Introduction

Study and control of plasmonic properties has become essential part of many current technologies^1,2^. At the nanoscale, light may be controlled in different ways by manipulation of surface plasmon polaritons (SPPs)^3^, which are collective oscillations of electrons at the interface between two materials^4–6^.This property has major consequences for diverse applications, including energy harvesting^7^, photodetection^8^and sensing^9^. Particularly in the field of two-dimensional (2D) materials like graphene and hexagonal boron nitride (h-BN), recent developments in nanofabrication and material science have enabled to explore innovative plasmonic materials and structures^10–12^.

Graphene/h-BN/substrate should be treated as a coupled heterostructure, where the visible-wavelength response is governed by the combined electrodynamic roles of all components rather than by any one material alone^13,14^. Graphene provides the conductive 2D layer whose induced currents and local near field determine the Raman response, h-BN acts as a wide-bandgap dielectric spacer and an edge-defining microstructure that controls near-field localization. The electromagnetic boundary conditions depend on the substrate by dielectric screening and optical losses processes, which have been identified to have a measurable influence on the vibrational and optical response of the supported graphene^15,16^. Past studies of G/h-BN heterostructures have characterized that the relative alignment as well as the dielectric milieu dominating medium affect Raman and optical expressions, thus supporting a heterostructures instead of single-material view^17^. More broadly, enhanced Raman scattering and strong near-field localization can also be achieved with dielectric resonators and nanostructures without relying on metallic plasmons, motivating wide-bandgap dielectric microstructures as low-loss field-shaping platforms^18^.

The capacity of graphene to sustain surface plasmon polaritons (SPPs) at terahertz to mid-infrared frequencies, which are highly controllable by chemical doping or electrostatic gating, is one of its most important properties^19,20^. The plasmons in graphene are tightly confined to the material’s surface, resulting in extremely high field enhancement, which is advantageous for applications in photodetection, light-matter interaction and sensing^21,22^. By dynamically varying the carrier concentration, the application of an electric field can further improve the plasmonic characteristics and enable real-time control of the plasmonic resonance^23^. Additionally, graphene can serve as an effective platform for nonlinear optical processes and quantum optics applications due to its ability to limit plasmons to sub-wavelength scales^24^. Graphene has been shown to be useful in tunable plasmonic devices, such modulators and switches, where optical stimuli or external electrical may dynamically affect the plasmonic resonance. These special qualities make graphene an ideal material for plasmonic and optoelectronic technologies of the future^25^.

Hexagonal boron nitride (h-BN), a wide-bandgap insulator with a honeycomb structure like graphene, is an essential material in the creation of plasmonic heterostructures due to its superior dielectric and chemical properties. In combination with other two-dimensional materials like graphene, h-BN acts as a perfect substrate or encapsulating layer, offering an atomically flat and neutral environment that increases plasmonic activity. This combination results in longer plasmon lifetimes and greater confinement by maintaining the high mobility of charge carriers in graphene and reducing plasmon damping^26^. In heterostructures, the relationship between graphene and h-BN produces hybrid plasmonic modes with unique properties. These modes gained from the h-BN layer’s strong confinement and decreased scattering losses, which greatly improves the heterostructure’s plasmonic response^27,28^. Furthermore, plasmonic resonances may be dynamically controlled by external electric fields that can be used to adjust the dielectric characteristics of h-BN. This tunability is very effective for the development of active plasmonic devices, including modulators and sensors, where real-time control over plasmonic characteristics is important^29,30^.

Silicon (Si) and silicon dioxide (SiO₂) are among the most widely used substrates in the field of nanophotonic and electronics, offering an adaptable structure that enables two-dimensional materials to be integrated like graphene^31^and h-BN^32^. Silicon can improve plasmon propagation and offer a way to integrate plasmonic components with electrical circuits, making it a useful platform for plasmonic devices, especially when paired with graphene^33^. High refractive index of Si helps to control the optical modes that are useful feature for effective photonic devices^34^. Moreover, the combination of Si and SiO₂ substrates with graphene/h-BN heterostructures allows the development of hybrid plasmonic-photonic devices that leverage the unique properties of both materials. This integration is crucial for advancing applications in sensing, photodetection, and on-chip optical communication.

Optical and electronic properties of heterostructures made of a high-quality single layer chemical vapor deposition (CVD) graphene laid over h-BN flakes on Si and SiO_2_ substrates were recently analyzed by micro-Raman spectroscopy mapping, Kelvin probe force microscopy, optical and atomic force microscopy^35^. Highly enhanced Raman intensity (up to 280%) from Si as well as from graphene as well as increased optical absorption along the G/h-BN edge was observed. It was attributed to localized concentration of electrons in graphene at the h-BN edge that gives rise to suitable perpendicular orientation of plasmonic vibrations at visible frequencies. This broadened the scope of otherwise infrared plasmonic features of graphene. The effect was specific to G/h-BN/Si structures, on G/*h*-BN/SiO_2_ structures the Raman signal was suppressed. Such electron accumulation features at the graphene/h-BN edge on Si substrate were corroborated also by scanning electron microscopy analysis^36^. However, theoretical understanding and corroboration of the observed effect with possibly broad scientific and practical implications is still missing.

Therefore, in this work, we perform physics-based theoretical simulations of G/h-BN heterostructures on Si and SiO_2_ substrates with particular focus on the edges, flakes, and surfaces of the G/h-BN heterostructure. The theoretical models of G/h-BN heterostructures on different substrates are designed and RF field distribution is simulated by finite element method. The effect of substrate material and thicknesses of graphene and h-BN layers on electrical field distribution and intensity is comprehensively studied. We provide a full-wave electromagnetic explanation of previously experimentally observed edge enhancement, and we explain on the theoretical basis the substrate “switch” behavior: strong edge enhancement on Si but strong suppression on SiO_2_. We show that already the simple RF field description is perfectly well describing the pronounced differences in plasmonic properties of G/h-BN heterostructures in different structural and material configurations. Moreover, we demonstrate that the same fundamental mechanism is not limited to h-BN and can be utilized with other wide-bandgap dielectric microstructures, so the results are useful beyond the specific G/h-BN case. We discuss the impact of these results on predicting suitable device design parameters and substrates and on explanation of prior experimental data. We provide a design map (dielectric thickness, graphene thickness, excitation wavelength) and identify the conditions for maximizing the edge hotspot, which can guide experiments and device designs.

## Model design and simulation method

**Fig. 1:**
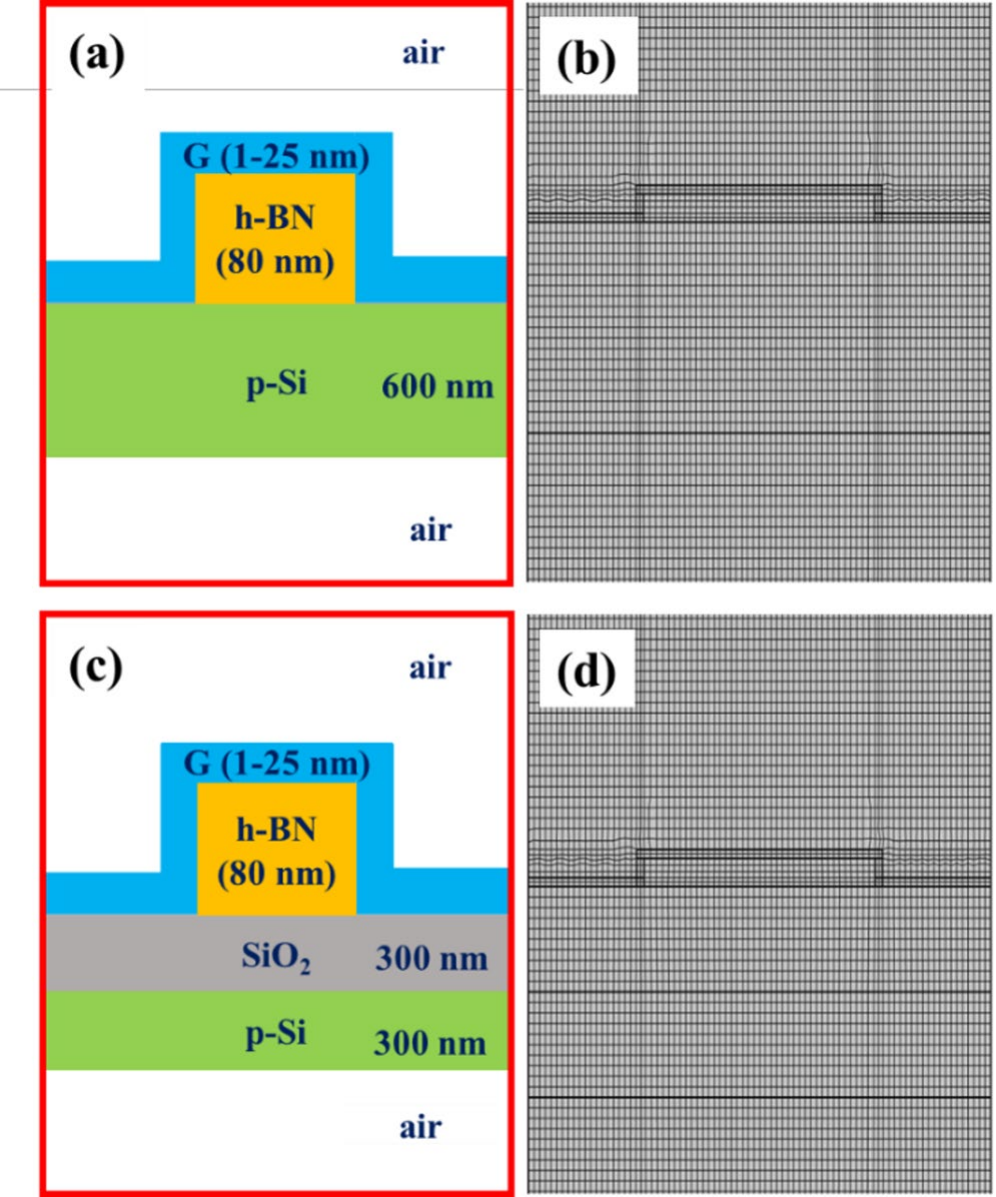
Structural and computational models of G/h-BN heterostructures: (a) Schematic view and (b) mesh for FEM simulations detailing the G/h-BN microstructure on p-type silicon substrate. (c) Schematic view and (d) mesh for FEM simulations detailing the G/h-BN microstructure on SiO_2_ substrate (SiO_2_ layer supported by p-type silicon wafer). Air is assumed as the surrounding medium in both cases.

Figure 1 shows the schematic design of two G/h-BN models on p-type silicon substrate with and without SiO_2_ buffer layer. The thicknesses of h-BN and graphene were parameterized, ranging in the simulations between 20 and 160 nm for h-BN and 1–30 nm for graphene, respectively, to analyze their impact on the heterostructure plasmonic properties. Graphene is implemented as a thin volumetric conductive layer. The electromagnetic effect of graphene is then dominated by the effective sheet conductivity of graphene, i.e. the integrated current response across the thickness. A 2D surface-conductivity model (e.g., Kubo-based surface current boundary) would be an alternative parameterization of the same sheet conductivity response. But it would not enable to characterize effects of graphene thickness, which increases the possible applicability of the study for various experimental situations and device designs with few layer graphene, multilayer graphene, graphene corrugations and alike. The overall width and height of the models were fixed at 1600 and 3000 nm respectively. The surrounding medium was defined as air. The structure was illuminated by a normally incident plane wave, with the electric field polarized in the plane of the 2D cross-section along the x-direction (Ex = 1 V/m, Ey = Ez = 0). The background electric field was set to 1 V/m. Scattering boundary conditions were imposed to avoid unwanted reflections from the surrounding. The wavelength of incident light was set to 632 nm based on the findings in Ref.^35^. Complementary analyses for the wavelength of 532 nm are provided in the Supplementary Information.

The radio frequency (RF) module in COMSOL Multiphysics was used for the study of designed 2D and 3D geometries. The RF module solves the full-vector, time-harmonic Maxwell equations considering also dispersions and losses. It is therefore applicable for plasmonic simulations at visible wavelengths. The validity of this approach in the optical regime relies on using appropriate complex material parameters at the operating wavelengths and sufficiently fine meshing to resolve the strong near-field gradients at graphene edges. The main limitations are those of a classical, local-response Maxwell model: it does not include quantum/nonlocal plasmonic corrections (e.g., nonlocal conductivity, spill-out, tunneling) that may become relevant only at sub-nanometer scales, so our results should be interpreted as classical field-localization and enhancement trends rather than a fully quantum plasmonic description.

The finite element method (FEM) was used to solve system of equations. The magnitude of electric field was estimated in three regions: edge, flake, and surface using identical region definitions in every simulation to enable direct, consistent comparison. For such numerical analysis, the geometry of the problem was discretized using a mesh. Definition of the finite element mesh is crucial for physical accuracy of the results. Various mesh types and mesh details were experimented with, until the results were independent of applied mesh and were free of mesh artifacts, in particular at sharp corners. At first, we tested the default physics-controlled mesh settings generated automatically by the simulation software, exploring various refinement levels such as fine, finer, extra finer, and extremely fine mesh. However, even for the extremely fine mesh we still observed a significant inconsistency in the computed electric field distribution, intensity values as well as in the thickness dependance. Abrupt variations indicated a problem with numerical stability and convergence, particularly in regions with high field gradients and complex geometry features. Such instability can be attributed to inability of automatic FEM meshing algorithms to generate sufficiently structured and conformal elements for the RF field simulations in critical regions of the multilayered nanoscale structures.

To address this issue, we tested an approach with user-controlled mapped meshing. The mapped meshing allowed for greater control over element distribution, especially across the layered interfaces and near the edge features where the field localization is most pronounced. A locally refined extremely fine mesh was used to accurately resolve the strong, nanometer-scale field gradients that occur near the graphene edge and interfaces. The employed refinement removes numerical inaccuracies, and ensures that the computed edge, flake and surface field values are stable so that they are not dominated by discretization artifacts.

It provided higher spatial resolution of electric field distribution and minimized discretization errors. The employed mapped mesh is illustrated alongside the models in Fig. 1. We verified across various heterostructure material and geometrical parameters that this approach provided stable and accurate electric field profile, particularly at the regions of interest, and ensured convergence of results across the mesh elements. As a result of this optimization, we employed an extremely fine mesh resolution within the mapped mesh framework as the optimal choice for all simulations that are presented here.

The key material properties for the RF field simulations are relative magnetic permeability, relative dielectric permittivity, and electrical conductivity. Properties for each material component of the model were specified considering typical data reported in the literature. They are summarized in Table 1. We also verified robustness of the simulations by testing variations of the material properties. For instance, silicon conductivity was varied by three orders of magnitude, which provided qualitatively and quantitatively similar results in terms of field distribution around the G/h-BN heterostructure.

**Table 1:** Relative magnetic permeability, relative dielectric permittivity, and electrical conductivity of the materials employed in the simulations.

Material	Properties		
	Relative permeability	Relative permittivity	Electrical conductivity
Graphene	1	5.8411	10^6^ S/m
Silicon	1	*ε*_*1*_ = 15.736 (Real) *ε*_*2*_ = 0.028734 (Imaginary)	10^3^ S/m
Silicon dioxide	1	2.1272	0
Hexagonal boron nitride (h-BN)	1	4.7649	0

The magnitude of electric field enhancement in the G/h-BN heterostructure model was evaluated in three specific regions denoted as the “flake”, the “edge” and the “vicinity”. Identical region definitions were used in every simulation to enable direct, consistent comparison. The evaluation concept is schematically shown in Fig. S1 in the Supplementary Information. The edge of the G/h-BN heterostructure was selected due to the pronounced Raman enhancement observed there experimentally. For comparison, the “flake” region is located on the graphene layer on top of the h-BN flake. The “vicinity” region is located outside the heterostructure, i.e. where graphene lays directly on the Si or SiO_2_ substrate. The very corners of the heterostructure were excluded from the evaluation, because there may be a concentration of electric field on such sharp asperities just from geometrical reasons. Thus, varying the corner geometry (e.g. rounded in practical devices) will not affect the observed trends and reproducibility. In all the three regions, the maximum field intensity was taken as representative. We considered also calculation of average values in the regions, but that did not provide correct information about the field enhancement due to the inherent inhomogeneous field distribution around the heterostructure.

The outside “vicinity” region was at first considered as the reference for electric field without the effect of h-BN. However, the initial simulations showed that the local electric field tends to spread quite far around the heterostructure, at least 500 nm in our model. The nearby outside region denoted as “vicinity” thus characterizes intensity of the RF field spreading in the microscopic surrounding of the G/h-BN heterostructure. This may actually help explain the spatial profile of Raman intensity across the heterostructure edge observed experimentally. However, it cannot be used as a true reference for RF field enhancement due to the heterostructure. Therefore, we have performed simulations with the planar models, where h-BN was removed completely and graphene was laid in plane directly on the Si or SiO_2_ substrate. The schematic models and respective simulation results are shown in Figs. S2 and S3 in the Supplementary Information. The obtained maximum electric field intensity at the graphene surface on such reference structures is then denoted as “background”.

## Results and discussion

### Heterostructures on silicon substrate

First, we analyzed how graphene thickness affects the electric field distribution and intensity. The thickness of graphene layer was varied from 1 nm to 25 nm with the step size of 5 nm for each case. The geometry and thickness of other materials were fixed. Thickness of h-BN was set to 80 nm based on prior experimental work showing the largest plasmonic effect around this thickness. For the Si substrate model (Fig. 1a) the p-Si substrate thickness was set to 600 nm.

**Fig. 2:**
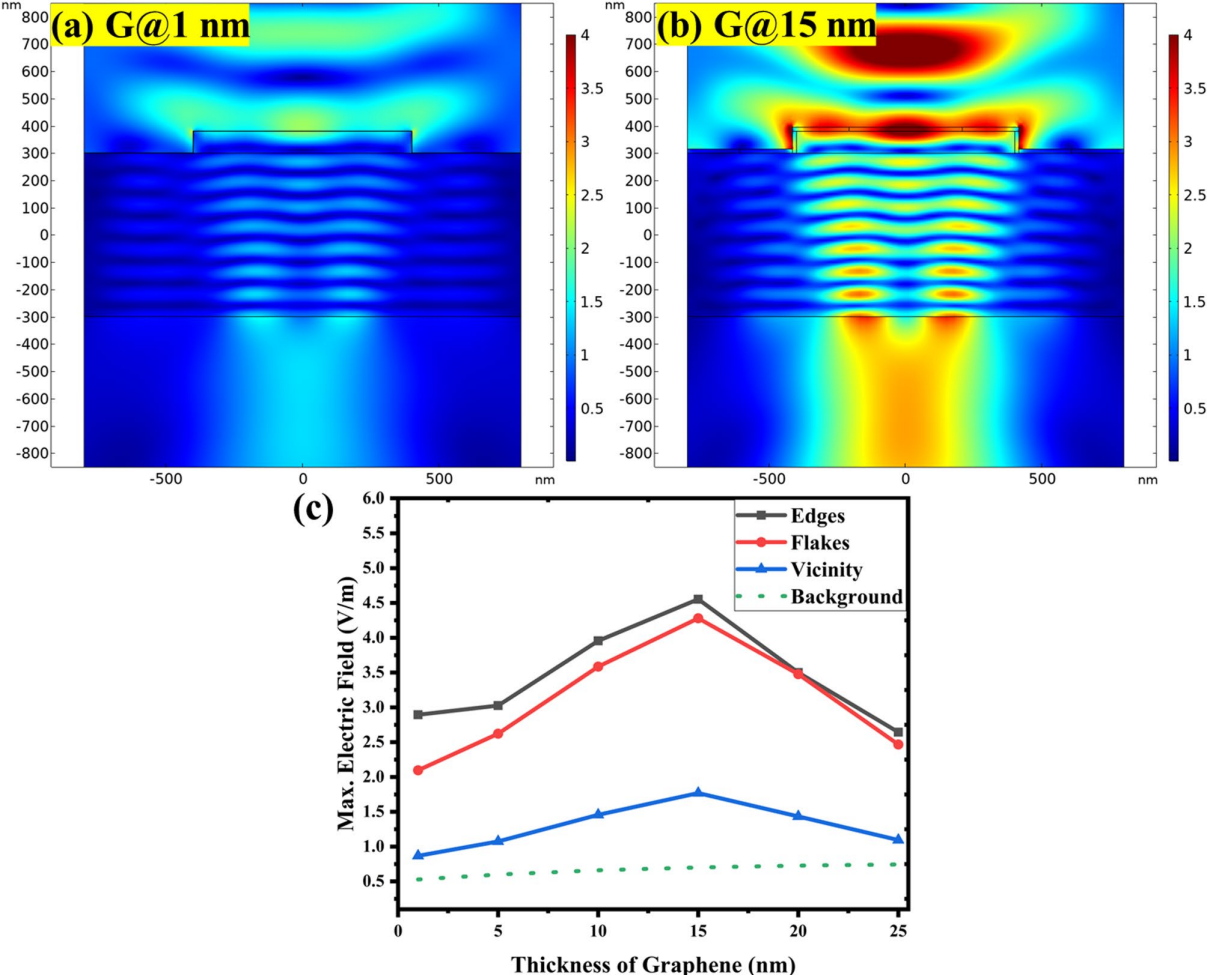
Analysis of electric field enhancement of G/h-BN heterostructure with 80 nm h-BN flake on Si substrate for different graphene layer thicknesses: Electric field intensity maps on the heterostructures with (a) 1 nm thin graphene layer and (b) 15 nm thick graphene layer, illustrating the localized field intensity distribution (color scale 4 V/m). (c) Quantitative plot of the maximum electric field as a function of graphene layer thickness with separate data sets for edge, flake, and outside surface area of the heterostructure and for the planar reference structure denoted as background.

Figure 2 shows the simulation results for different graphene thicknesses laid over 80 nm h-BN layer on Si substrate. It provides electric field intensity maps on the heterostructures with 1 nm and 15 nm graphene layer, illustrating similarity in the localized field intensity distribution. At the same color scale it is obvious that the electric field is concentrated at the edge of heterostructures in both cases. Only for the larger graphene thickness the field appears focused along the larger extent of the edge. Actual values of electric field intensity evaluated from the edge, flake and outside vicinity area (by using procedure described in the Methods section) are summarized for graphene thickness from 1 nm to 25 nm in the graph in Fig. 2c.

The trend as function of thickness is not monotonous, with maximum at around 15 nm. Within the whole thickness range, the most pronounced field enhancement is at the edge of heterostructure, reaching up to 10 times more than the background reference value. Even for only 1 nm graphene there is about 6 times field enhancement at the h-BN edge. The focused field in such a thin layer bend over the edge may facilitate most pronounced charge carrier accumulation compared to more bulky material^37,38^. A single layer of graphene will also help avoid strong interlayer and substrate coupling of plasmonic modes. It may be the key features for the experimentally observed plasmonic enhancement under visible frequency excitation.

The h-BN thickness was varied from 20 nm to 160 nm to analyze its impact on the electric field distribution and enhancement. The presence of h-BN represents not only structural support for graphene but it can be helpful to control the electronic properties of graphene^31^. These electronic properties can be tuned by adjusting the h-BN thickness. The graphene layer thickness was fixed at 5 nm for being close to real experimental single layer with some topography modulations and for yielding high field enhancement at the edge and on the flake compared to the outside surface as shown in the Fig. 1c. The substrate parameters were the same as above.

**Fig. 3:**
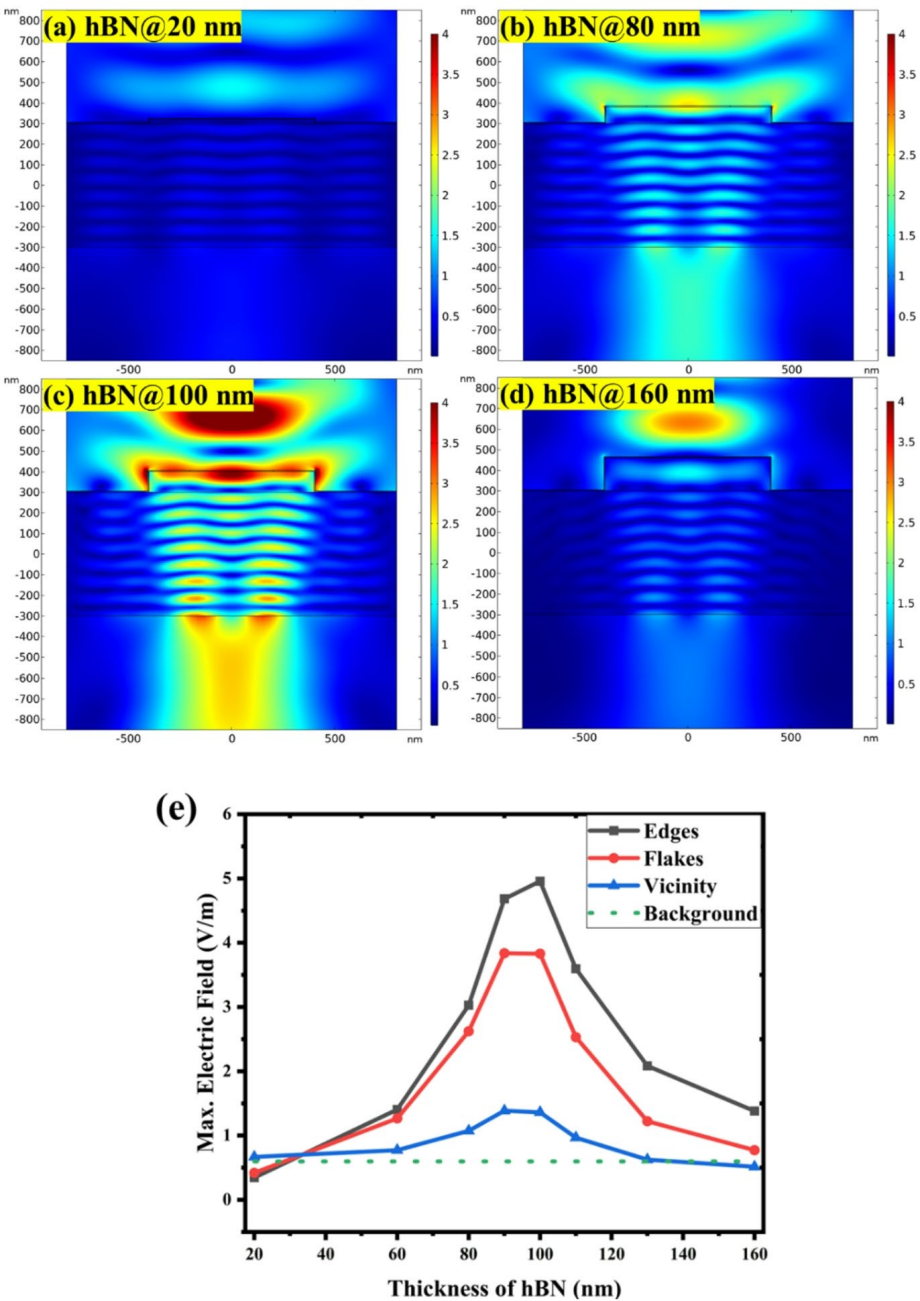
Analysis of electric field enhancement of G/h-BN heterostructure with 5 nm graphene for different h-BN flake thicknesses on Si substrate: Electric field intensity maps on the heterostructures with (a) 20 nm, (b) 80 nm, (c) 100 nm, and (d) 160 nm thickness of the h-BN flake, illustrating the localized field intensity distribution (color scale 4 V/m). (e) Quantitative plot of the maximum electric field as a function of h-BN flake thickness with separate data sets for edge, flake, and outside surface area of the heterostructure and for the planar reference structure denoted as background.

The results of these simulations for the 5 nm graphene and varied h-BN thickness on Si substrate are shown in Fig. 3. It provides electric field intensity maps on the heterostructures with 20 nm to 160 nm h-BN flake layers, illustrating pronounced changes and differences in the localized field intensity distribution. It is obvious that the electric field is localized at the heterostructure edges with high intensity only for the medium h-BN thickness around 80–100 nm. The simulations also show that the electric field is oriented perpendicularly at the edge, which may be crucial feature for the experimentally observed plasmonic effects.

There are also pronounced variation of electric field distribution and intensity within the h-BN flake itself. Although there is some increased field intensity on the flake compared to outside area, in particular for some h-BN thicknesses, it is lower than at the edge. This is in a good agreement with experimentally obtained Raman maps on such heterostructures^35^.

Actual values of electric field intensity evaluated from the edge, flake and outside areas are summarized for h-BN thickness from 20 nm to 160 nm in the graph in Fig. 3c. The graph confirms the non-monotonous field enhancement at the G/h-BN edge as function of h-BN thickness. There is some but weaker enhancement also on top of the G/h-BN flake. These findings suggest that the edge plays a key role and that the plasmonic enhancement is optimal in G/h-BN/Si structures with specific thickness parameters, providing a pathway for achieving maximum Raman signal enhancement in practical applications. The highest electric field is found at the edge around 80–100 nm h-BN thickness. This optimum is in striking agreement with the experimental Raman spectroscopy observations on such samples^35^. This means that the present model based on RF field analysis and simple material parameter set and structural model represents a sufficiently accurate model for the experimentally observed plasmonic effect at the G/h-BN edge on Si substrate. Raman scattering process can be boosted by this local field enhancement, since the Raman scattering cross-section scales with the fourth power of the local electromagnetic field enhancement.

The h-BN thickness dependance of the electric field on the outside surface area exhibits interestingly similar albeit lower profile as at the h-BN edge. The reason is that the field localization at the edge is spreading far enough (~ 200 nm as observed in the maps) to influence electric field intensity evaluation on the outside surface. The reach of plasmonic field maybe farther in such structures, which may have practical benefits. This may also help explain the spatial profile of Raman intensity across the heterostructure edge observed experimentally^35^.

Compared to all above effects of the G/h-BN heterostructure, the “background” electric field on the planar graphene structure on Si substrate (see the model and results in Fig. S2) is generally an order of magnitude smaller than on the G/h-BN heterostructures and field focusing at the graphene surface is missing. This directly confirms the pronounced effect of the G/h-BN heterostructure on the field enhancement.

### Heterostructures on SiO_2_ substrate

**Fig. 4:**
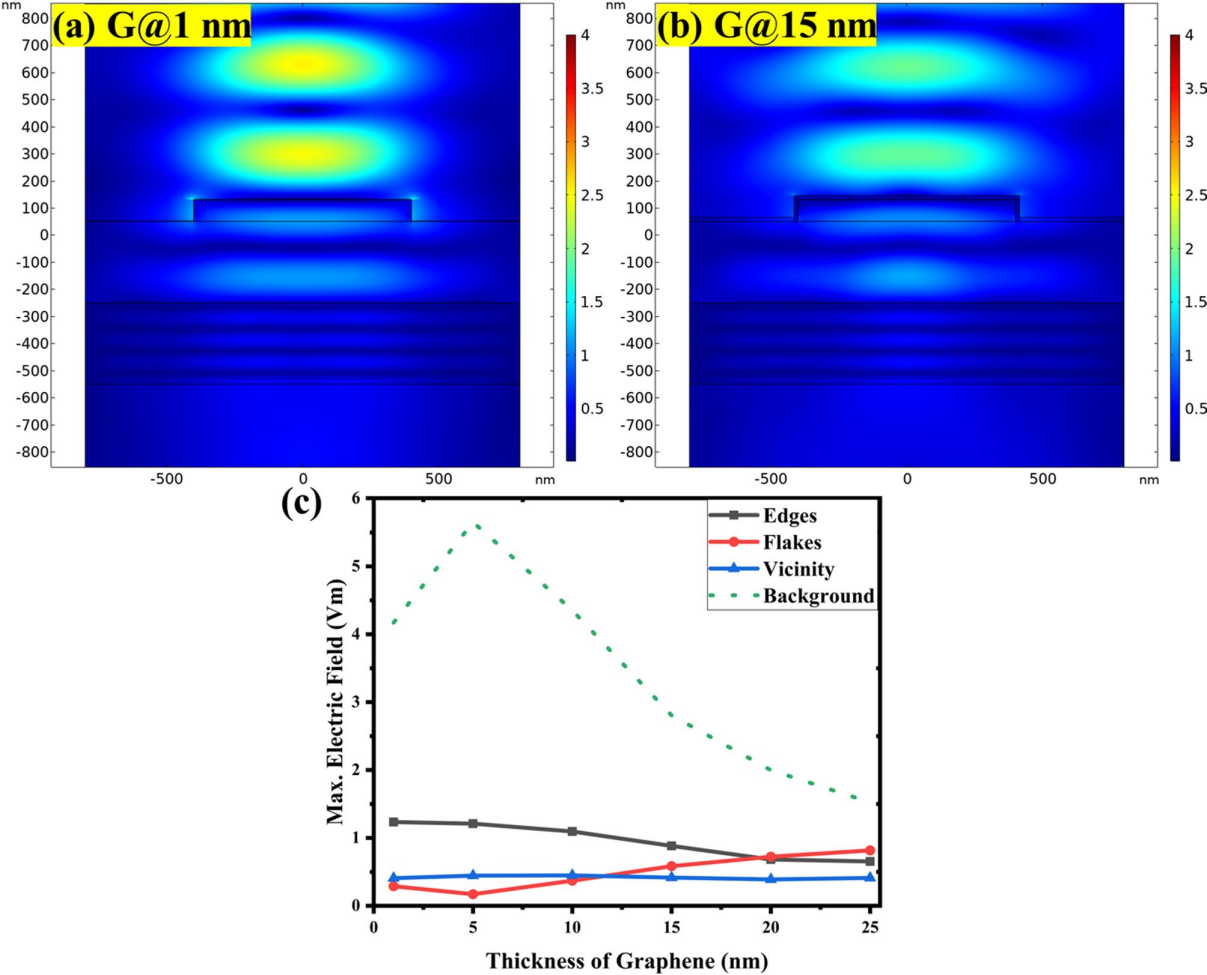
Analysis of electric field enhancement of G/h-BN heterostructure with 80 nm h-BN flake on SiO_2_ substrate for different graphene layer thicknesses: Electric field intensity maps on the heterostructures with (a) 1 nm thin graphene layer and (b) 15 nm thick graphene layer, illustrating weak field intensity distribution (color scale 4 V/m). (c) Quantitative plot of the maximum electric field as a function of graphene layer thickness with separate data sets for edge, flake, and outside surface area of the heterostructure and for the planar reference structure denoted as background.

The results in Fig. 4 show the results of simulations with variable graphene thickness in the case of the SiO_2_ substrate. For the SiO_2_ substrate model (Fig. 1c) the thickness parameters were SiO_2_ = 300 nm, p-Si = 300 nm. The effect of the thickness of graphene layer on electric field distribution and intensity at the h-BN edges, on top of the h-BN flakes and on the outside area of the G/h-BN heterostructure is again plotted as maps and evaluated. Unlike the results on the Si substrate in Fig. 3, the electric field maps look very different. Also, graphene thickness seems to have hardly any impact. The intensity of the electric field is weak overall. There are no focused hotspots at the h-BN edges.

Actual values of electric field intensity evaluated from the edge, flake and outside area are summarized in Fig. 4c. Indeed, the electric field intensity is about and order of magnitude lower overall, compared to the values on the Si substrate in Fig. 2c. Electric field intensity at the edge or on the flake is similar and follows similar weakly decreasing trend for increasing graphene thickness. Thus the field enhancement effect at the G/h-BN edge is largely suppressed by the use of SiO_2_ substrate. The insertion of SiO_2_ layer has thus tremendous impact on both the field distribution and focusing as well as intensity. This is again in strikingly good agreement with the experimental Raman spectroscopy observations on such system^35^. It corroborates validity of the employed physical model and at the same time it confirms the suggested plasmonic enhancement mechanism behind the experimental observations on Si substrates.

**Fig. 5:**
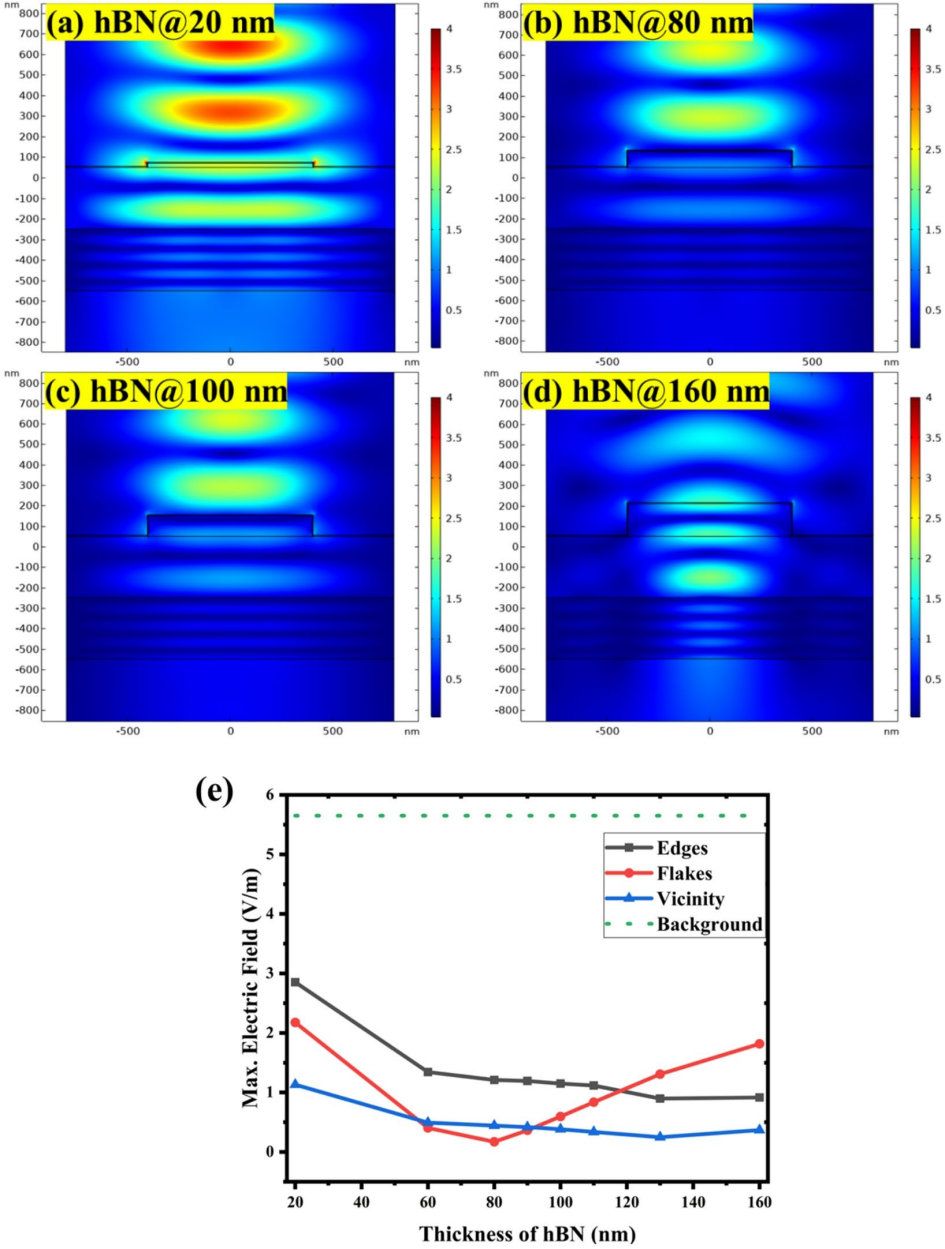
Analysis of electric field enhancement of G/h-BN heterostructure with 5 nm graphene for different h-BN flake thicknesses on SiO_2_ substrate: Electric field intensity maps on the heterostructures with (a) 20 nm, (b) 80 nm, (c) 100 nm, and (d) 160 nm thickness of the h-BN flake, illustrating the localized field intensity distribution (color scale 4 V/m). (e) Quantitative plot of the maximum electric field as a function of h-BN flake thickness with separate data sets for edge, flake, and outside surface area of the heterostructure and for the planar reference structure denoted as background.

Figure 5 shows the results of simulations for the 5 nm graphene and varied h-BN thickness on the SiO_2_ substrate. Electric field intensity maps on the heterostructures with 20 nm to 160 nm h-BN flake layers illustrate pronounced changes and differences in the localized field intensity distribution. The electric field becomes localized at the heterostructure edges towards the low h-BN thickness of 20 nm. There are also pronounced electric field distribution and intensity variations within the h-BN flake itself. The field localization thus seems to arise from a suitable electric field intensity interference at particular to h-BN thickness.

Actual values of electric field intensity evaluated from the edge, flake and outside area are summarized in Fig. 5c. The overall electric field intensity is well below the background reference and it is also weaker than on the Si substrate in Fig. 3. All the dependencies exhibit similar decreasing trend for increasing h-BN thickness towards 100 nm. Somewhat higher electric field at the edge is between the flake and the background field, making thus monotonous intensity trend across the edge. These theoretical findings are again in perfect agreement with experimental observations on G/h-BN/ SiO_2_ heterostructures at lower h-BN thickness, where no edge enhancement and lowering Raman signal was observed^35^.

For the higher h-BN thicknesses the flake profile starts to increase. This is agreement with the electric field maps and corroborates the field interference explanation. Experimentally, the high thickness of h-BN flakes (60 nm or more) were not available on the SiO_2_ substrate. This theoretically predicted effect may thus be explored in some further experimental works.

The results in Fig. S3 in the Supplementary Information show that the “background” electric field on the planar structures on SiO_2_ substrate is generally of higher magnitude than on the heterostructures and a field resonance is localized close to the graphene surface. This is well correlated with the experiment^35^where the G/h-BN heterostructure suppresses the Raman signal compared to the outside surface. This again confirms the pronounced effect of the G/h-BN heterostructure on the field focusing and enhancement, in particular at the heterostructure edge on the Si substrate.

The various graphene thickness 5 nm was selected as an effective thickness within the finite-element implementation to represent graphene as a conductive film in a volumetric formulation, accounting for its possible corrugations or multilayers in practical devices. To assess the effect of a single layer, we performed also simulations using 0.3 nm graphene layer under the same geometry and excitation conditions for both Si and SiO2 substrates (Fig. S8 in the Supplementary Information). The resulting field map confirms that the edge-localized field enhancement at the G/h-BN step remains clearly present at single layer thickness in the case of Si substrate. This shows that the core mechanism is not dependent on assuming a thicker graphene layer. The thickness choice mainly affects the quantitative magnitude through the effective sheet response.

### Excitation wavelength dependance

**Fig. 6:**
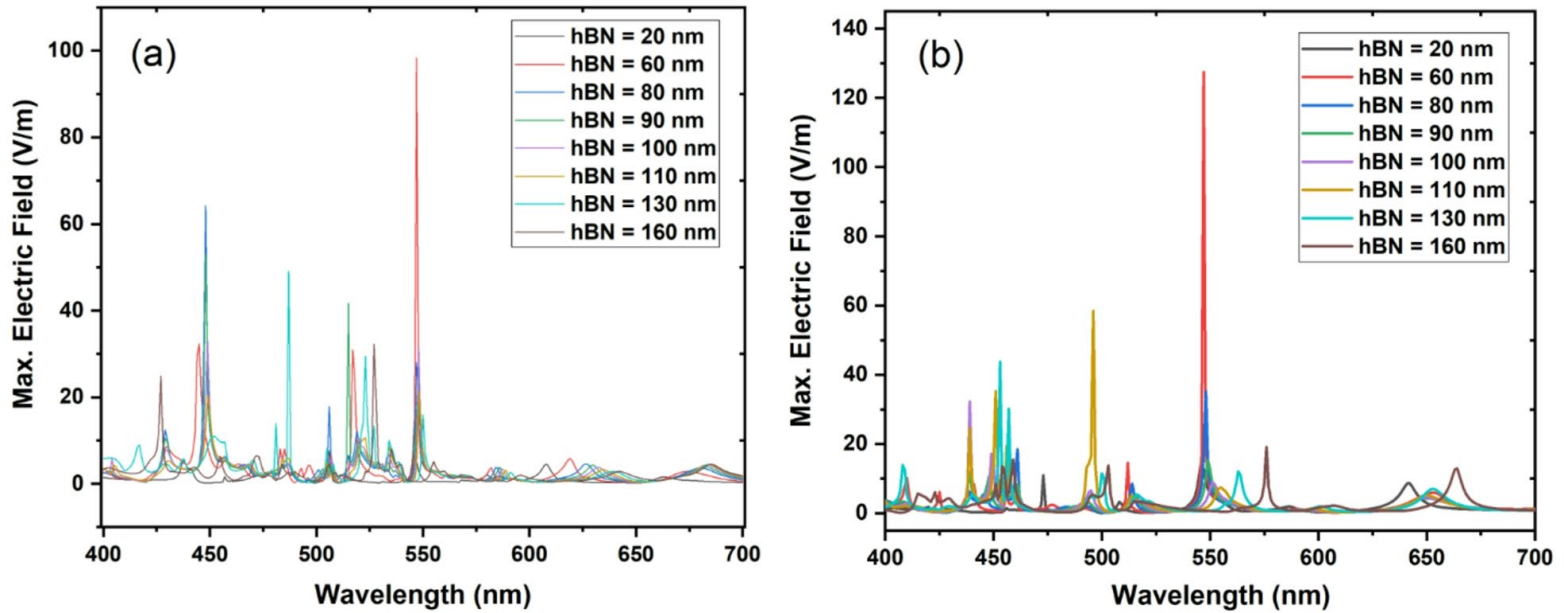
Spectral dependance of the electric field at the G/h-BN edge on (a) Si substrate, (b) SiO_2_ substrates.

The full visible range spectra of the maximum electric field intensity as function of h-BN thickness in Fig. 6 predict that the most pronounced effect of the field enhancement could be obtained at 550 nm–450 nm. This prediction may serve as motivation for future Raman experiments on the G/h-BN heterostructures, where the laser, notch filter and other components of the setup must be modified to enable reliable Raman spectroscopy using these excitation wavelengths.

All the simulations were thus performed also at another experimentally relevant excitation wavelength of 532 nm. The results for G/h-BN heterostructures on both Si and SiO_2_ substrates are shown in Figs. S4-S7 in the Supplementary Information. Also, at this wavelength the heterostructures exhibit similar effects of enhanced field localization at the edge as described above, which corroborates the robustness of the simulations. Resonances are obvious not only for the model with Si substrate but also for SiO_2_ substrate. This is discussed further below in section “Broader context and predictions”.

### Three-dimensional model

The simulation models and results presented so far have been designed as two-dimensional cross-sections through the G/h-BN structure on Si or SiO_2_ substrates. Thereby the simulations were computationally less expensive and enabled to explore and validate various observations. Already in the 2D representation the simulations were in a perfect agreement with experimental observations in the literature^35^. Nevertheless, to obtain more comprehensive view and to further validate the physical mechanisms occurring, the full three-dimensional (3D) model has been designed. The case of G/h-BN/Si heterostructure was selected for the 3D model as it shows the most pronounced field enhancement and G/h-BN edge field localization, which is prospectively the most application relevant.

**Fig. 7:**
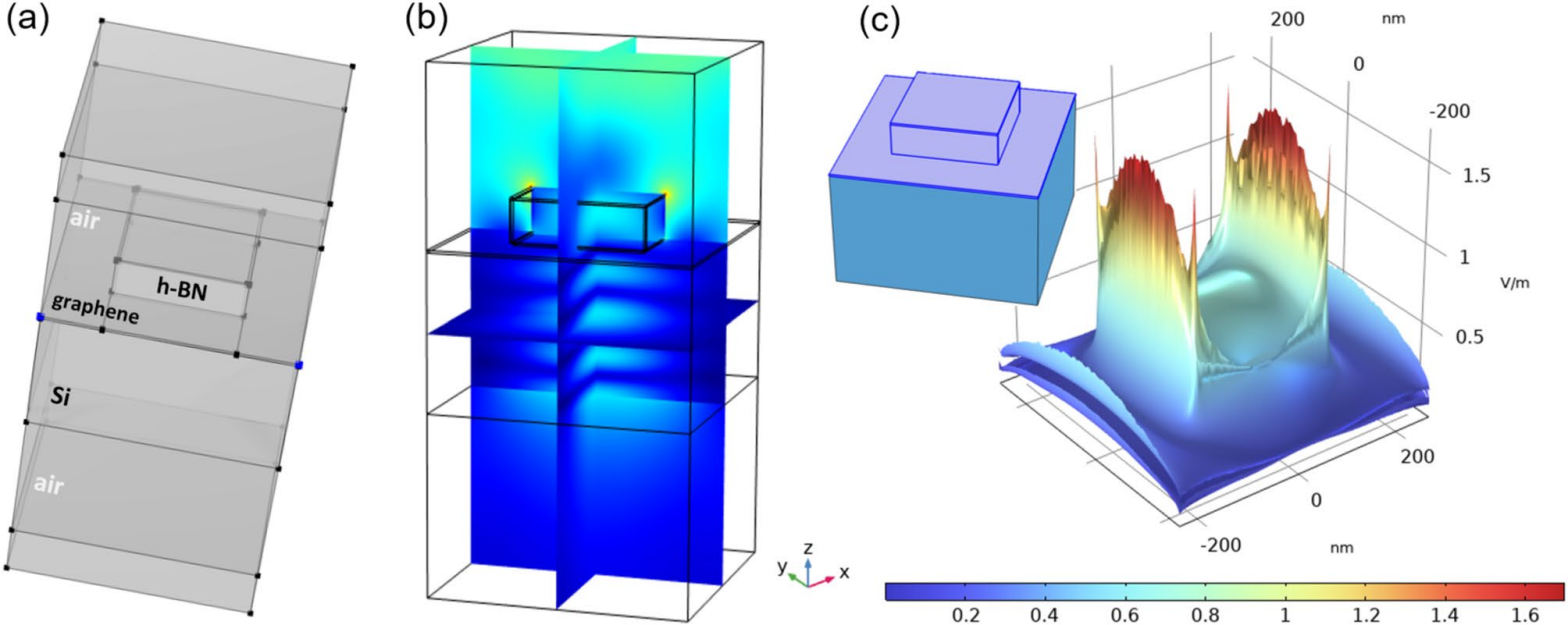
(a) 3D model of G/h-BN/Si heterostructure with 80 nm h-BN flake confined on the surface and full surface overlay by 5 nm of graphene. (b) Electric field intensity maps in the cross-sections along the major perpendicular directions under two viewing angles. (c) Comprehensive 3D electric field distribution visualization around the G/h-BN heterostructure.

Figure 7 shows outline of such 3D model of G/h-BN/Si heterostructure with 80 nm h-BN flake confined on the surface and full surface overlay by 5 nm graphene. The simulations were done with a full 3D mesh. The results are shown in Fig. 7b under various angles, where electric field intensity maps are shown as cross-sections along the major perpendicular directions. Figure 7c also shows full 3D field distribution visualizations by using COMSOL post-processing routines.

The 3D model fully corroborates the significant focusing and enhancement of RF electrical field at G/h-BN flake edge. It can be seen in the cross-sectional maps as well as in the full 3D visualization. Thus, the model physics is also in 3D in full agreement with actual experimental observations on real samples with three-dimensional structures.

In detailed view, one may notice that field focusing at the h-BN edge is lower in the perpendicular cross-section. It is not an artifact but the effect of real physics. While the simulations solve full-wave Maxwell equations leading to multidirectional field propagation, the incident RF field is polarized horizontally. As a result, the horizontal cross-section captures stronger field confinement due to alignment with the excitation polarization. This gives rise for instance to typical asymmetric field around gold or silver nanoparticles^39^. In real experiments with non-polarized light, and enhancement along the whole edge would be expected and it was indeed observed^35^. On the other hand, using a polarized light may be a way for the field enhancement at particular place or direction, which may be interesting for both physical-chemical studies and some applications.

### Broader context and predictions

The RF field simulations are commonly used for simulation of plasmonic effects on conductive materials. The RF field distribution is governed mainly by a complex dielectric constant of the materials, including the metals. In the simulations here, we indeed observed the field focusing also on h-BN structures without graphene for some h-BN heights on Si or SiO_2_ substrate. Raman scattering process could be in principle boosted by this local field enhancement, since the Raman scattering cross-section scales with the fourth power of the local electromagnetic field enhancement. However, although dielectric materials with their positive permittivity play a crucial role in supporting and shaping the plasmonic phenomena, there have hardly any free electrons to actually exhibit plasmon resonances. Some increase in Raman signal may also be possible due to optical effects such as light incoupling and outcoupling or interference. However, the spatial optical, structural and Raman profiles showed that merely optical effects cannot explain the edge enhancement^35^. Moreover, the difference between Si and SiO_2_ substrate observed in the simulated RF field intensity across the G/h-BN heterostructures, where at a same excitation wavelength on the same G/h-BN heterostructure the Si substrate provides enhancement and SiO_2_ substrate does not, is in perfect agreement with the prior experiments^35^. Conductive substrate obviously represents another prerequisite for free electron transfer into graphene into these focused regions, thereby giving rise to localized surface plasmons in graphene in the visible spectral range. We can thus assume that the field intensity enhancement and focusing observed in the simulations will support electron localization in the graphene that is laid over such dielectric structures. Multi-physics simulations combining RF, wave optics and semiconductor modules may provide further insight into these phenomena^40^.

The doping of graphene can principally alter the excitation wavelength conductivity of conductive graphene. When graphene would be more or less conductive, the strength of the edge hotspot may become stronger or weaker and the optimum conditions (e.g. the maximum value itself and the optimum wavelength) may vary. In our study, the key effect of strong edge field enhancement on Si within an optimal h-BN thickness window (and suppression on SiO_2_) is mainly controlled by the substrate boundary condition and the h-BN geometry. Thus, the graphene doping (e.g. via charge transfer from Si substrate) or gating can serve as another tuning parameter that modulates the scale of the enhancement.

The estimation of Raman scattering from the local enhanced field is in principle straightforward. Raman enhancement ~ ∣E_max_ / E_0_​∣^4. In our case E_0_ = 1 V/m and thus Raman enhancement ~ ∣E_max_ ​∣^4. However, it is only proportionality factor. Actual Raman scattering intensity depends also on the derivative of polarizability α with respect to vibrational direction. Graphene or silicon polarizability can vary depending on actual material conditions and it is frequency dependent. Thus, calculating relevant Raman intensity values for reasonable comparison with experiments remains rather challenging.

**Fig. 8:**
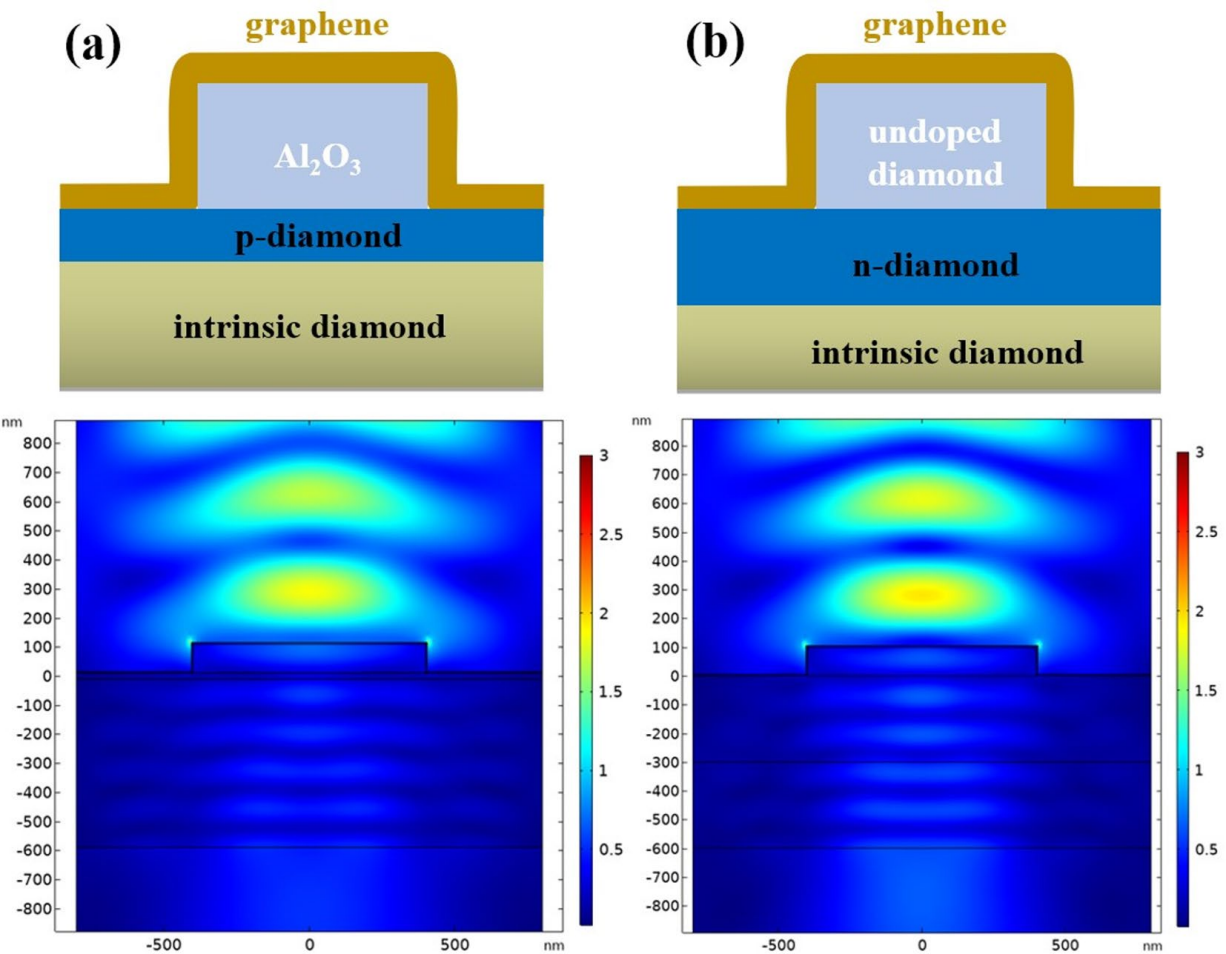
Analysis of electric field distribution of heterostructure models where graphene is laid over two other types of microstructures: (a) p-type surface conductive diamond layer with 100 nm Al_2_O_3_ mesa structure, (b) n-type diamond layer with 100 nm undoped diamond mesa structure. Graphene thickness 5 nm. Electric field intensity color scale 3 V/m.

The simulation models and methodology established above enable also to explore in-silico a possible use of materials other than h-BN flakes for generating localized visible frequency plasmons and corresponding Raman scattering enhancement. For instance, CMOS compatible materials would facilitate better control and broader use of such graphene-based plasmonic structures. As the first step in this direction, preliminary simulations of two types of structures were performed using the same methodology. Models and results of these preliminary simulations are shown in Fig. 8. The first type of structure is p-type surface conductive diamond layer with Al_2_O_3_ mesa structure. The second type is n-type diamond with undoped diamond mesa structure. In accordance with the above results on G/h-BN heterostructures, both mesa structure height was set to 100 nm, graphene thickness was 5 nm and total substrate thickness was 600 nm, where p-type surface conductive layer on intrinsic diamond was set to 20 nm while n-type doped diamond layer was set to 300 nm. The field distribution around both heterostructures exhibits similar edge focusing features as for G/h-BN system. The simulation results thus predict that the diamond-based heterostructures may act in the same way for visible frequency plasmon generation as the G/h-BN/Si heterostructures. This provides incentives for practical fabrication and experimental measurements of plasmonic Raman enhancement in such graphene-diamond heterostructures.

## Conclusion

A comprehensive computational analysis of plasmonic field enhancement in graphene/hexagonal boron nitride (G/h-BN) heterostructures on Si and SiO_2_ substrates was performed. Finite element method simulations of radio-frequency electromagnetic fields demonstrated that the electric field distribution, local focusing and intensity are highly sensitive to both the structural configuration and material composition of the heterostructure. Notably, the simulations revealed a pronounced electric field enhancement at the G/h-BN edge for h-BN thicknesses between 80 and 100 nm on silicon substrates, in excellent agreement with prior experimental Raman spectroscopy data. In contrast, the presence of a SiO₂ substrate significantly suppressed the field localization and intensity, showing the critical role of substrate conductivity in enabling visible frequency plasmonic effects in graphene. The simulations further predict optimal excitation wavelengths and provide insight into the directional nature of field confinement, as confirmed by three-dimensional modeling. Beyond graphene/h-BN systems, the modeling approach was extended to diamond and Al_2_O_3_ microstructures, predicting that similar edge-localized plasmonic enhancement can be achieved with CMOS compatible materials that have more practical industrial relevance. These findings thus establish a robust theoretical framework for understanding and tailoring plasmonic behavior in graphene in visible light frequencies. The study thus may open new avenues for the design of tunable, substrate-engineered graphene plasmonic devices for sensing, optoelectronics, and photonic integration. Future simulations and experimental research can explore these interactions under various measurement settings or surrounding conditions such as water or vacuum, which may provide further insight into the observed phenomena and may be relevant for specific applications.

## Supplementary Information

Below is the link to the electronic supplementary material.

Supplementary Material 1

## Data Availability

The data related with the manuscript are available upon request from the authors or online from the Zenodo repository https://doi.org/10.5281/zenodo.16761121.
