# Supplementary material for: Computational analysis of visible frequency plasmonic properties of graphene on wide band gap heterostructures
Source: Sci Rep. 2026 Feb 15;16:9138. doi: 10.1038/s41598-026-40039-y (PMC12996562; doi:10.1038/s41598-026-40039-y)
Supplement: Supplementary file 1 — Supplementary Material 1 [file 41598_2026_40039_MOESM1_ESM.pdf]

SUPPLEMENTARY INFORMATION

Computational Analysis of Visible Frequency Plasmonic Properties of Graphene on Wide Band Gap

Muhammad QAMAR[1], Ghulam ABBAS[1], Meiyong LIAO[2], Satoshi KOIZUMI[2], Takatoshi YAMADA[3], Bohuslav REZEK[1*]

[1] *Faculty of Electrical Engineering, Czech Technical University in Prague, Technická 2, 16627 Prague, Czechia*

[2] *National Institute for Materials Science (NIMS), 1-1 Namiki, Tsukuba 305-0044, Japan*

[3] *National Institute of Advanced Industrial Science and Technology (AIST), Central 5, 1-1-1 Higashi, Tsukuba 305-8565, Japan*

*corresponding author: rezekboh@fel.cvut.cz

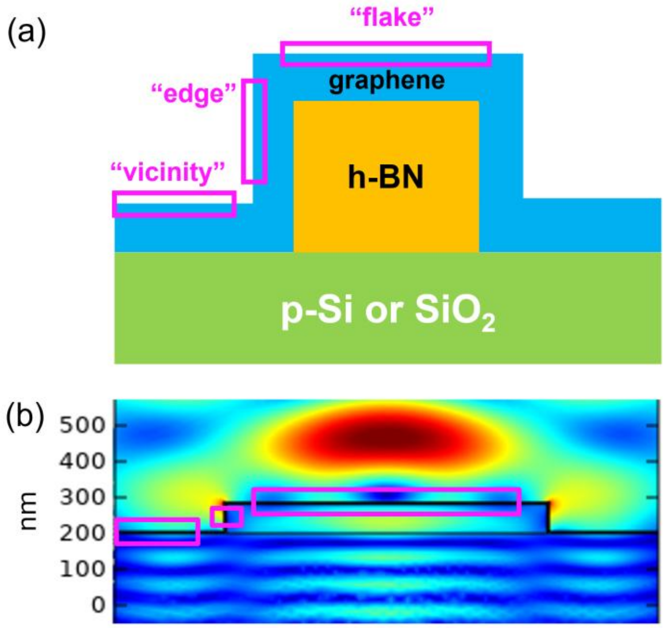

Figure S1: (a) *Schematic concept of three regions on the G/h-BN heterostructure, in which electric field intensity is evaluated. They are denoted as the vicinity, the edge, and the flake. The corners of the heterostructure are excluded by purpose as the electric field may be concentrated on such sharp asperities just from geometrical reasons. (b) Illustration of the three evaluation regions on the actual G/h-BN heterostructure model with 3 nm graphene, 80 nm h-BN and p-Si substrate.*

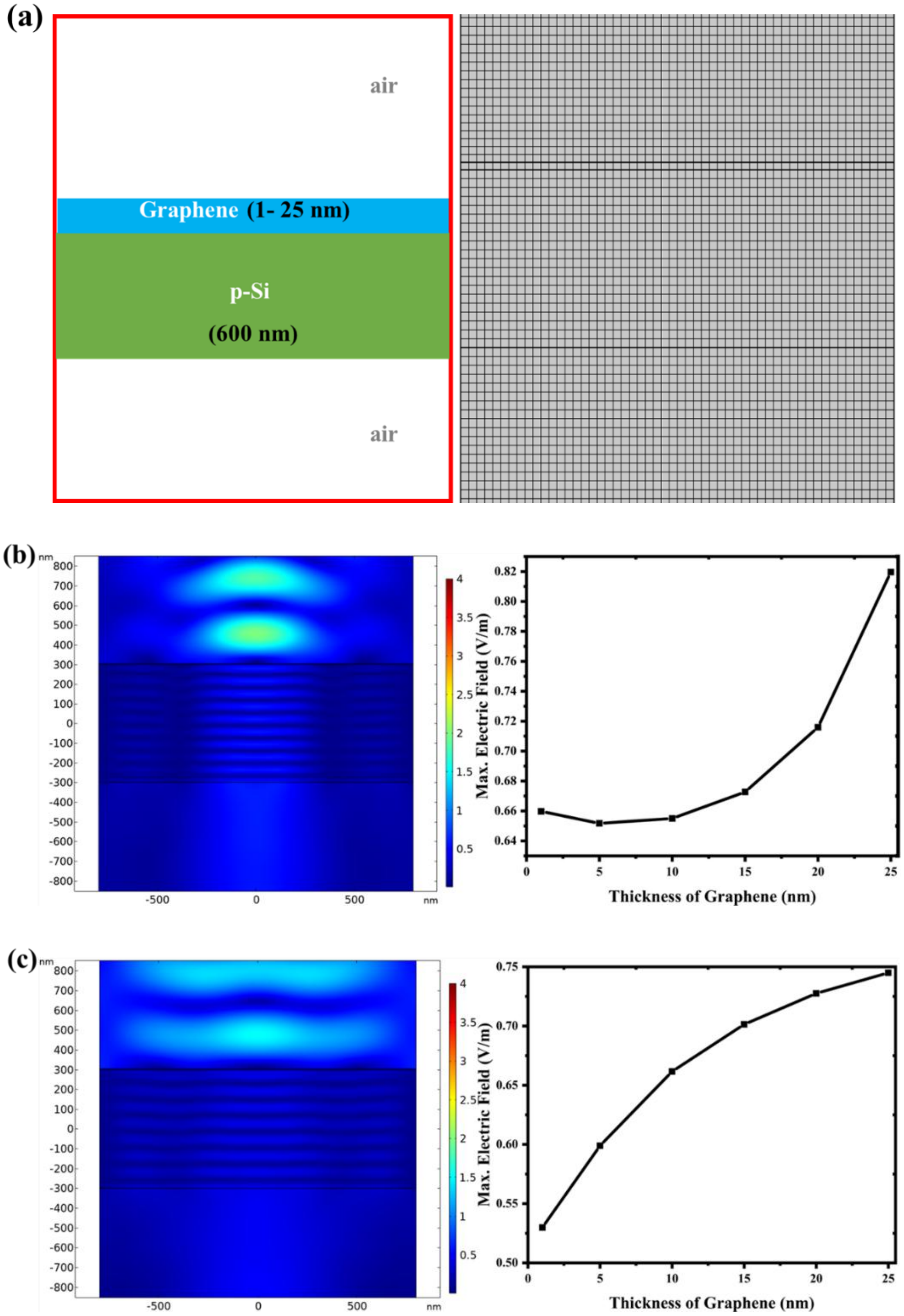

Figure S2: *(a) The schematic drawing and (b) mapped ultrafine mesh of the planar models, where h-BN is removed completely and graphene is laid in plane directly on the Si substrate. Respective simulation results of the planar models: (c) RF field intensity distribution map (color scale 4 V/m) for graphene thickness 5 nm and (d) maximum field intensity on the graphene surface plotted as function of the graphene thickness for plane wave illumination from top with (b) 532 nm wavelength and (c) 632 nm wavelength. The value at graphene thickness of 5 nm is used as the field "background" reference for comparison with the results on G/h-BN heterostructures.*

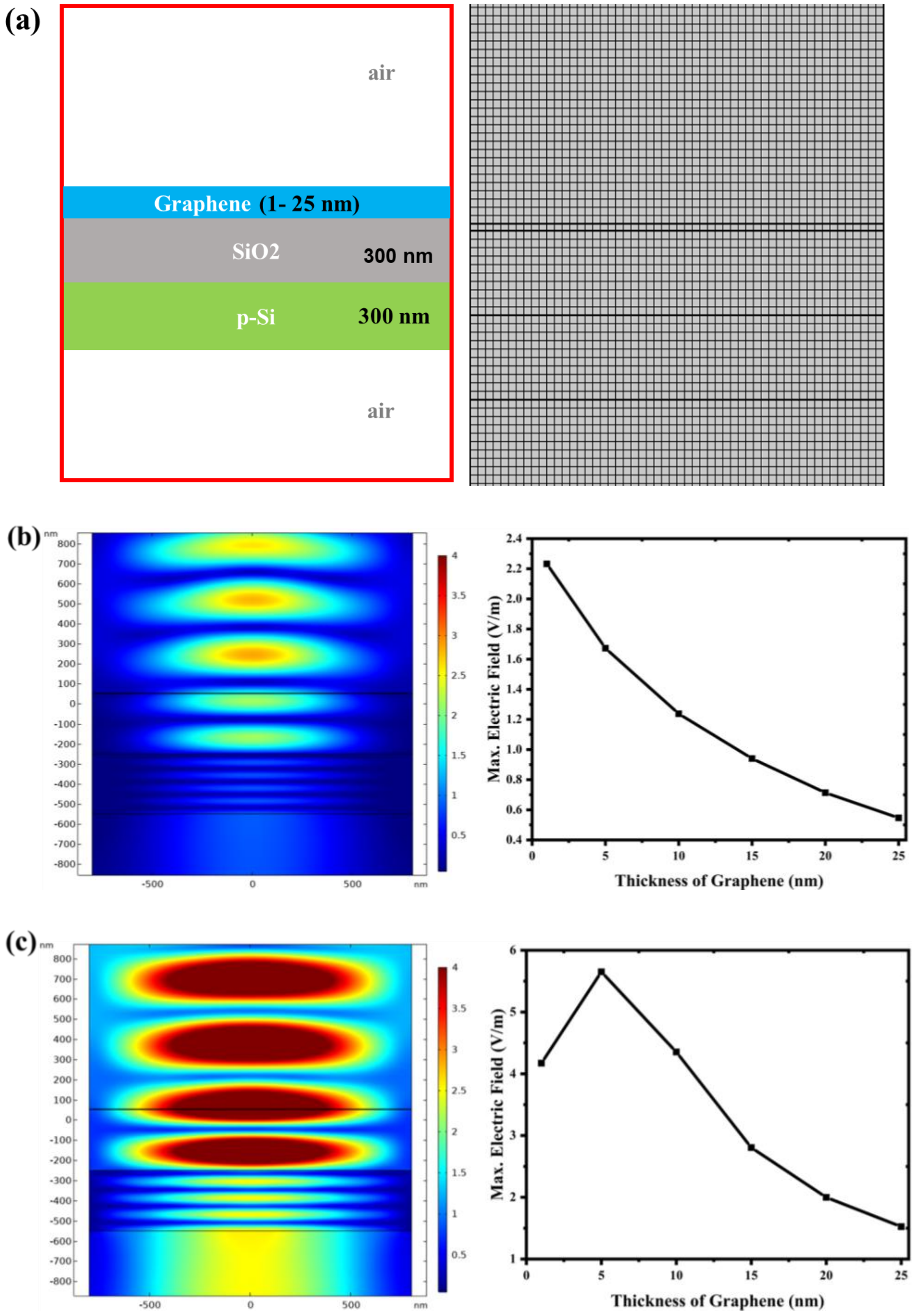

Figure S3: *(a) The schematic drawing and (b) mapped ultrafine mesh of the planar models, where h-BN is removed completely and graphene is laid in plane directly on the SiO_2 substrate. Respective simulation results of the planar models: (c) RF field intensity distribution map (color scale 4 V/m) for graphene thickness 5 nm and (d) maximum field intensity on the graphene surface plotted as function of the graphene thickness for plane wave illumination from top with (b) 532 nm wavelength and (c) 632 nm wavelength. The value at graphene thickness of 5 nm is used as the field "background" reference for comparison with the results on G/h-BN heterostructures.*

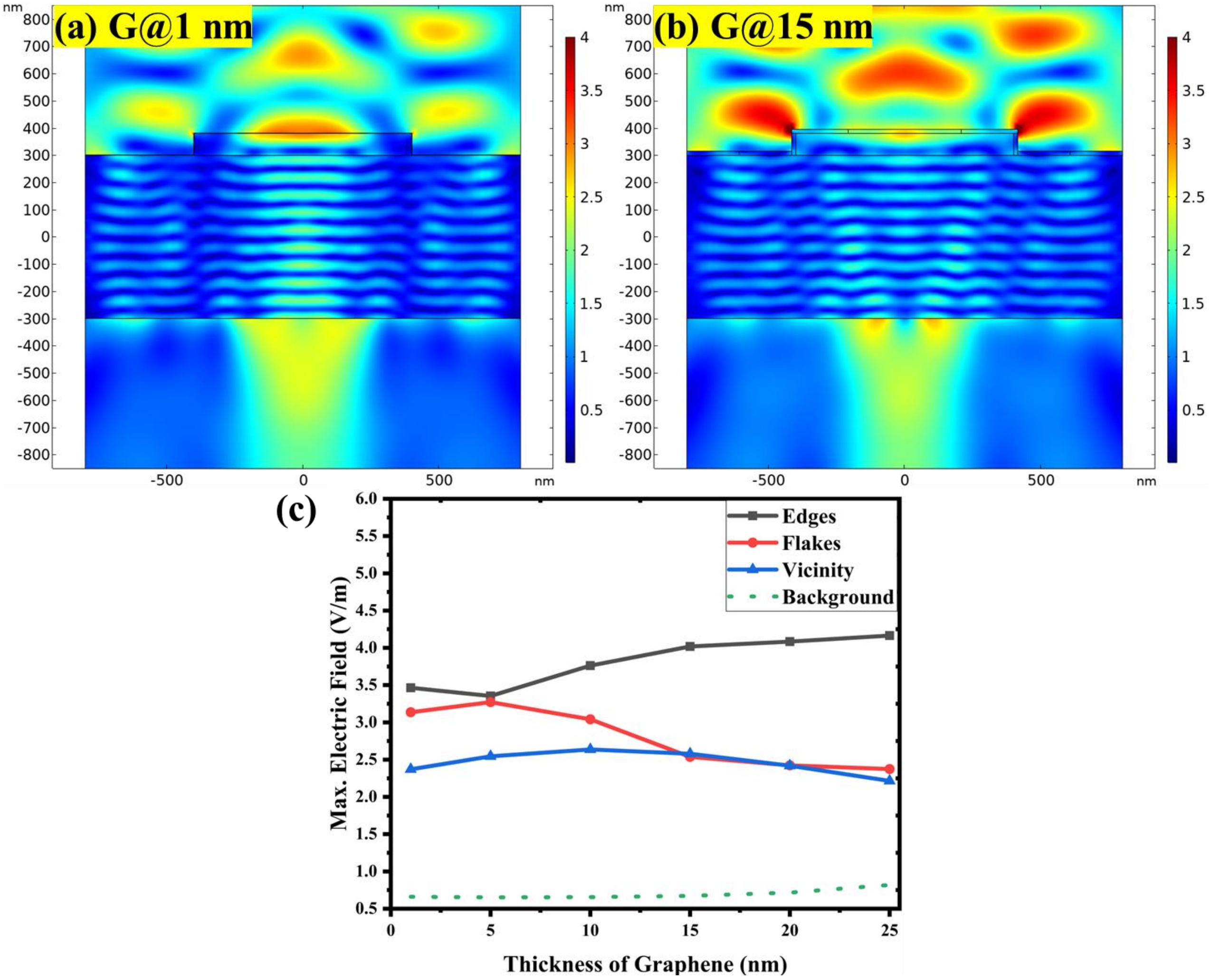

Figure S4: *Analysis of electric field enhancement of G/h-BN heterostructure with 80 nm h-BN flake on Si substrate for different graphene layer thicknesses: Electric field intensity maps on the heterostructures with (a) 1 nm thin graphene layer and (b) 15 nm thick graphene layer, illustrating the localized field intensity distribution (color scale 4 V/m). (c) Quantitative plot of the maximum electric field as a function of graphene layer thickness with separate data sets for edge, flake, and vicinity of the heterostructure and for the planar reference structure denoted as background. Incident illumination wavelength was 532 nm.*

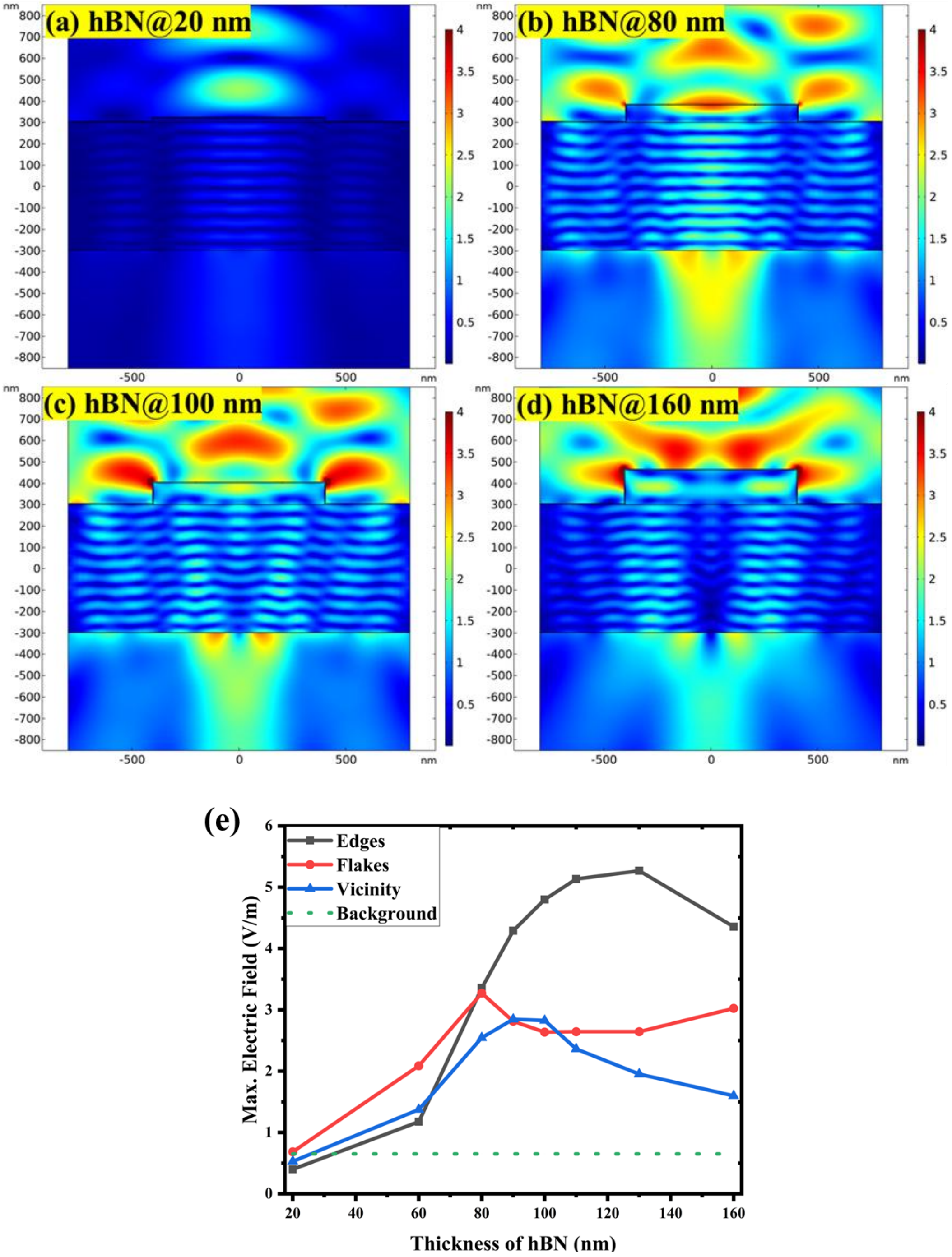

Figure S5: *Analysis of electric field enhancement of G/h-BN heterostructure with 5 nm graphene for different h-BN flake thicknesses on Si substrate: Electric field intensity maps on the heterostructures with (a) 20 nm, (b) 80 nm, (c) 100 nm, and (d) 160 nm thickness of the h-BN flake, illustrating the localized field intensity distribution (color scale 4 V/m). (e) Quantitative plot of the maximum electric field as a function of h-BN flake thickness with separate data sets for edge, flake, and vicinity of the heterostructure and for the planar reference structure denoted as background. Incident illumination wavelength was 532 nm.*

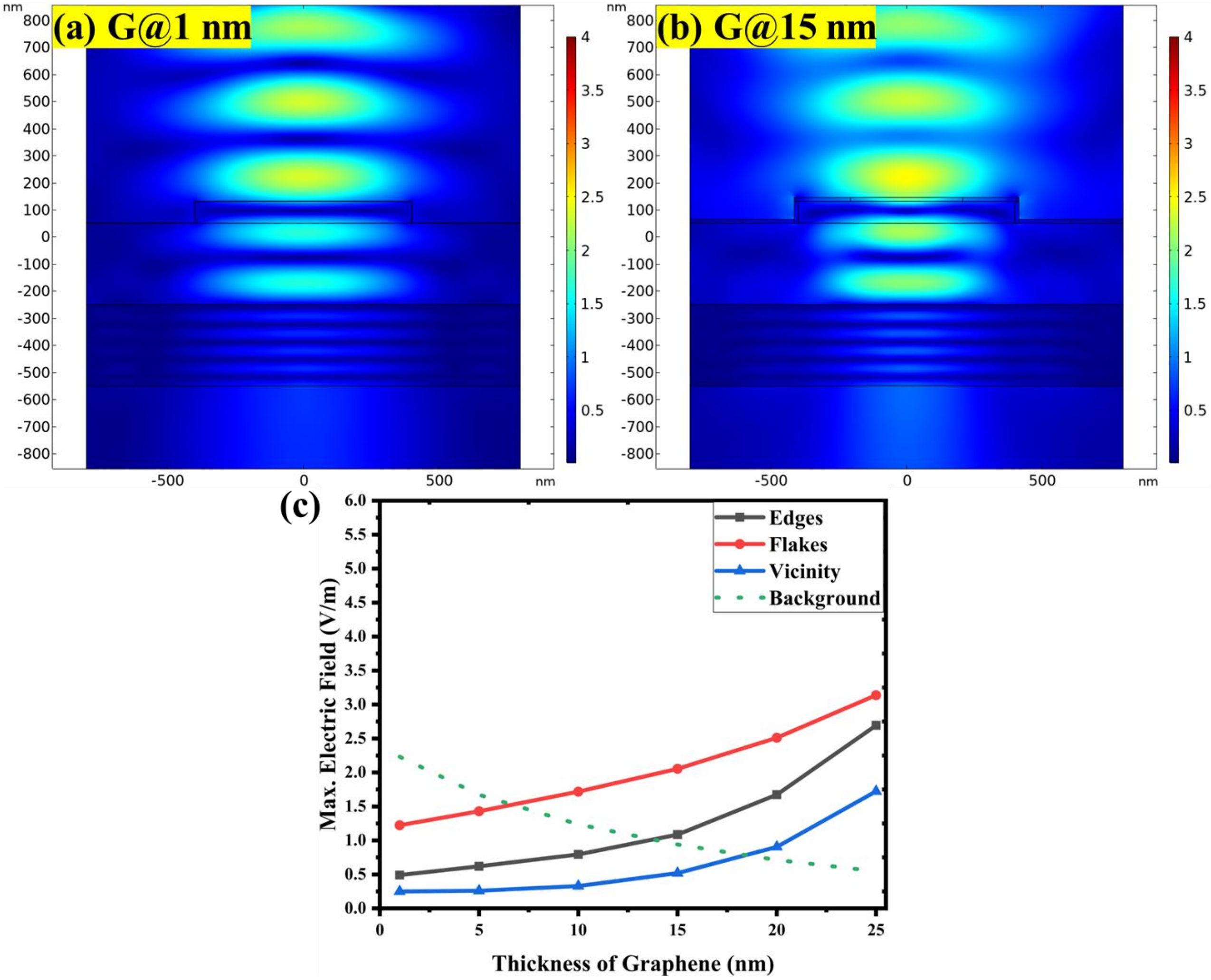

Figure S6*:* *Analysis of electric field enhancement of G/h-BN heterostructure with 80 nm h-BN flake on* SiO_2 *substrate for different graphene layer thickness: Electric field intensity maps on the heterostructures with (a) 1 nm thin graphene layer and (b) 15 nm thick graphene layer, illustrating weak field intensity distribution (color scale 4 V/m). (c) Quantitative plot of the maximum electric field as a function of graphene layer thickness with separate data sets for edge, flake, and vicinity of the heterostructure and for the planar reference structure denoted as background. Incident illumination wavelength was 532 nm.*

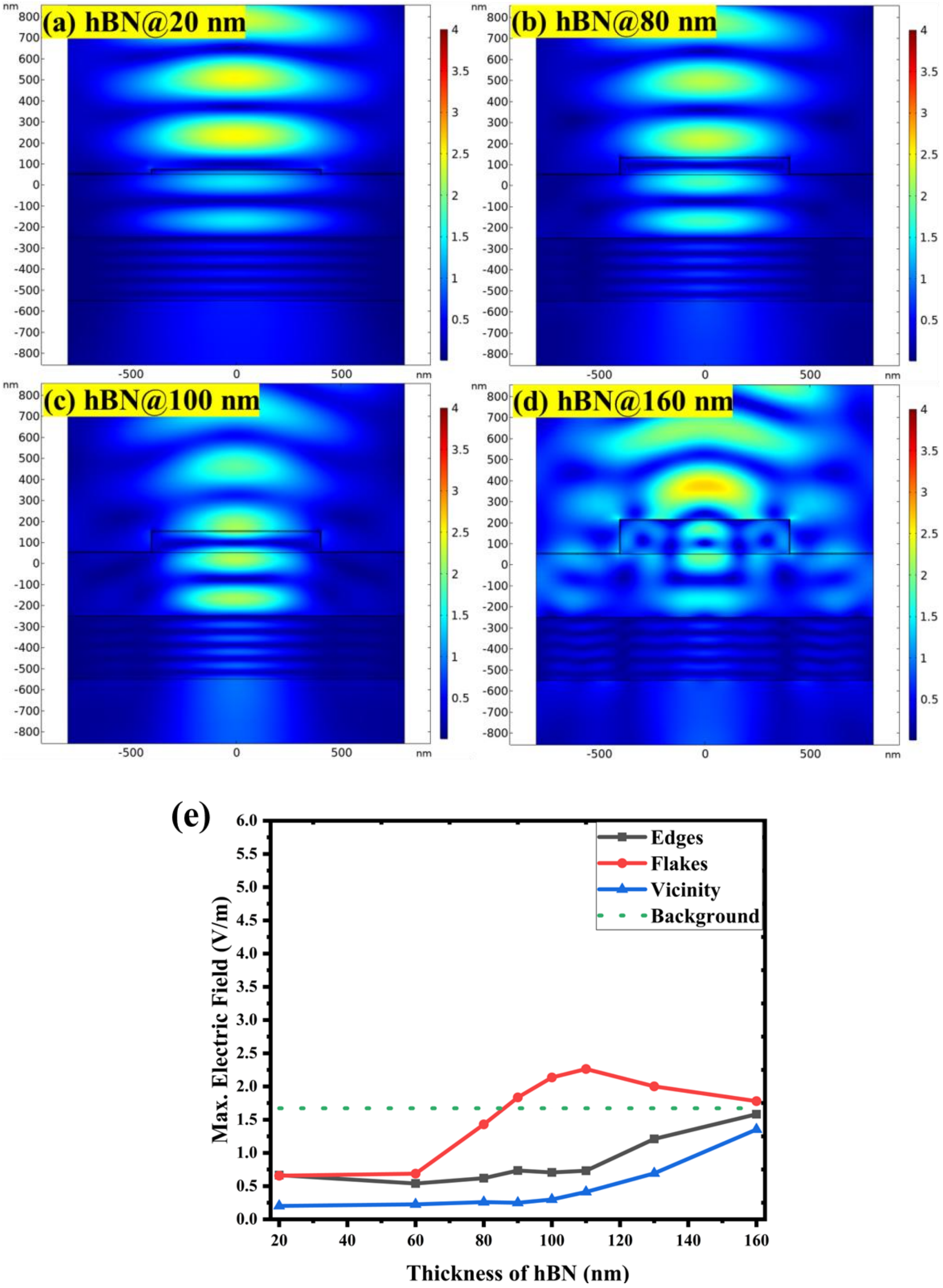

Figure S7*:* *Analysis of electric field enhancement of G/h-BN heterostructure with 5 nm graphene for different h-BN flake thicknesses on* SiO_2 *substrate: Electric field intensity maps on the heterostructures with (a) 20 nm, (b) 80 nm, (c) 100 nm, and (d) 160 nm thickness of the h-BN flake, illustrating the localized field intensity distribution (color scale 4 V/m). (e) Quantitative plot of the maximum electric field as a function of h-BN flake thickness with separate data sets for edge, flake, and vicinity of the heterostructure and for the planar reference structure denoted as background. Incident illumination wavelength was 532 nm.*

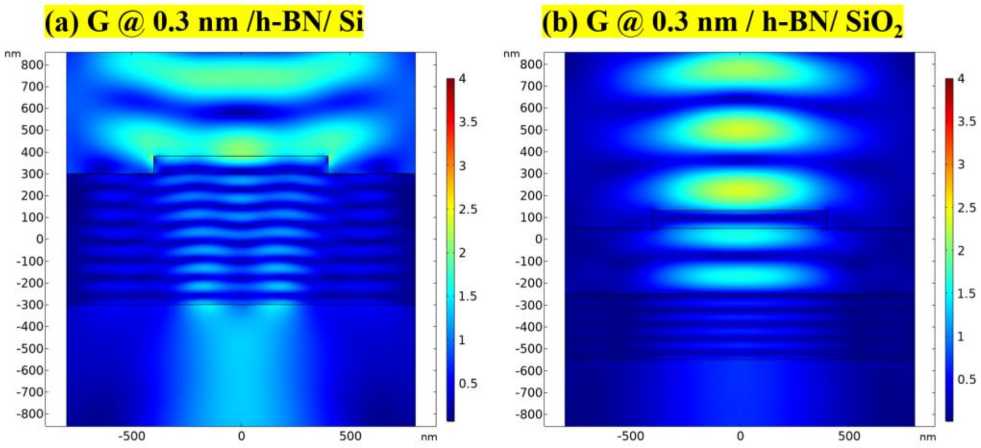

Figure S8: *Simulated electric-field plot for a monolayer-scale graphene layer (t = 0.3 nm) on 80 nm h-BN microstructure on (a) Si substrate, (b)* SiO_2 *substrate.*
